# Report of Cases in Obstetrical Practice

**Published:** 1858-01

**Authors:** Thomas Bevan


ARTICLE III.

[DECEMBER MEETING.]

### REPORT OF CASES IN OBSTETRICAL PRACTICE.

READ BEFORE THE COOK CO. MEDICAL SOCIETY.

BY THOMAS BEVAN, M. D.

Case 1.—Mrs. S——n, aged 22 years, in good health, married two years, has had two miscarriages. On the evening of Friday, Nov. 13th, accompanied her mother to the railroad depot. On returning home about dusk, she encountered a man, who made indecent proposals to her. She was very much frightened, although he did not attempt to forcibly detain her in his presence. She got out of his way as soon as she could, and made the best of her way home. Her husband noticed she was looking pale and excited, but she went about her work as usual, getting tea; notwithstanding, she felt some pain in the lower part of the abdomen, and from its intensity she was obliged to stop and sit down once or twice before she had finished. She ate but little, and after supper lay down on the lounge. Her pains came on at intervals, and increased in poignancy and intensity, until about ten o'clock p.m., when feeling a desire to evacuate the bowels, she passed in the vessel what proved to be a fœtus, between four and five months advanced. I was then called; found her in some pain, and the after-birth not yet delivered.

It was got away without difficulty, however, and with the exception of considerable nervous excitement, tendency to weep and complain rather more than usual, seemed to be doing well. I ordered a full dose of morphine to be repeated in four or five hours, if she was not quiet. Called the next morning, found my patient had had a good sleep, and was very comfortable.

I thought this case worth reporting, because it seems to be one, so far as I am able to judge, where miscarriage was brought on by the effect of a strong mental emotion. The miscarriages she had had previously were not so far advanced as this, and she was taking more than usual care to avoid that accident. She went on to convalescence without any unusual occurrence, and is now well.

CASE 2.—Mrs. A——n, aged 30 years, the mother of eleven children, married when she was thirteen years of age, lives in the second story of a house in State street, having the backstairs outside of the building, Nov. 10th, in going down stairs she slipped, and in her effort to avoid falling struck the abdomen against the hand rail. She felt a little sick at the stomach at the time, but the symptoms passed off and she thought no more of it. In two or three days she noticed a discharge from the vagina unlike what she had been accustomed to, with rather a fœtid odor; not troubling her much, and no alarming symptom arising, she did not consult a physician. At about three o'clock, the morning of the 20th, I was called and found her in labor, with the head of a child engaged in the inferior strait, which after an hour or two of moderately expulsive effort was born. It was dead, and no effort at resuscitation was instituted—the surface presenting the general palor that exists after life has been extinct for several hours. On passing my finger up the cord, I found another child engaging in the mouth of the womb, which after a few minutes was expelled. This last was also dead, and presented the appearance of having been dead eight or ten days. The cuticle was detached in several places; the odor was that of decomposition, and there was a purplish discoloration of the general integument; the liquor amnii was very fœtid, and not abundant; the cord of the first child was three or four times as large as that of the second, indicating that absorption had already commenced, for it is not probable that simple arrest of development for eight or ten days would account for the difference in size, even when coupled with the accidental difference usual in twin births. This woman was between the eighth and ninth months of gestation.

CASE 3.—Mrs. K——bs, in good health, pregnant with her fifth child, consulted me about three months ago for a discharge from the vagina, usually of a yellowish color and quite abundant, that had existed six or seven days; sometimes she had a litttle pain, and occasionally the discharge was sanguinolent. I recommended her to keep as quiet as possible, as the discharge was most abundant when she was on her feet, and told her I thought it was the ammotic fluid escaping, and that it would increase the chances of miscarriage. An examination at this time did not reveal anything more than some increase of the moisture of the vagina and a softened condition of the os; no appreciable dilatation. On the night of Nov. 21st, at twelve o'clock, her husband came to me, saying the child was born, and wanted me to see his wife, who was feeling very faint, immediately. I found the child was born, and the mother flowing considerably, with a small rapid pulse, and the after-birth not yet delivered. I removed it as soon as I could, and induced contractions by frictions and a snug bandage and compress.

The following is the history of her labor: She awoke suddenly from a quiet sleep in great pain, and rousing her husband, before he could dress her child was born, having been accomplished in just three pains, according to her story, neither more or less. It is probably impossible for labor to go through all its phases to the birth of the child in three pains, so that this must have been a case in which the first stage or that of dilatation was so gradual and painless as to fail to rouse her. There was very little water discharged at the time of her delivery; the woman felt nothing of the usual gush of waters during its progress. The child seemed between seven and eight months advanced and lived till the second morning, seeming to die exactly as children do from general bronchitis. It had lain an hour in a room without fire, and it is probable, if it had been kept thoroughly warm all the time, might have lived. This case seems to demonstrate that a hard labor does not always occur where there has been premature escape of the waters, though they seem to have exerted some influence in bringing on miscarriage.

I report these two last cases, that they may be added, if worthy of it, to the number of cases where the waters have passed off prematurely, and the comparative frequency of miscarriage following it; also, the length of time a woman may carry twins, one being dead from an external cause, in an advanced state of pregnancy, and its general effect upon the life of the other fœtus.

Case 4.—Concerns more directly the child than the mother. Mrs. C——h, a healthy woman, married two years, first child, had a good labor and is doing well. This lady had her menses last March 15th, which, if we count from the middle of her menstrual month, would make the fœtus eight months, but it is probably nearer term. The child was born alive. Its movements were not active, however, and after the nurse had washed the anterior part of the body, turning it over, she found the appearance you observe [specimen exhibited to the society] at the lumbar region of the spine. I examined it, and established the existence of a spina bifida, extending from the dorsal vertebræ to the sacrum. The tumor presented a deep purplish or florid appearance, and the external envelope was a very delicate shining membrane, seeming to be an extension of the skin, but very much thinned and altered in its characters. I explained to the husband its nature, and dressed it with a soft piece of old linen, imbibed in oil, and a cotton compress, retained in place by the usual bandage, so as slightly to compress it. There was not much vitality about the little fellow, although he drank some sugared water, evacuated the bowels, and passed the urine. It lived from ten o'clock p.m., on Wednesday, until five o'clock p.m., on Thursday. Its cry was weak, respiration irregular, and there was during the whole time a very congested condition of the head and face; the fontanelles were not unusually prominent, and moderate pressure on the tumor did not seem to determine any accident to the child.

Spina bifida, as far as I am aware, is rather a rare deformity in this country, according to *Ollivier*, *Dict. de Med.*, *Art. Hydrorachis*, vol. 16. Chaussier reports of 132 cases of malformation of various kinds occurring or deposited at the Maternite out of 22,293. Twenty-two were spina bifida, or about one in 1000. The influence of this vice of conformation on fœtal life is not very noxious, for according to authors, children born with this malady are generally alive, but after birth it is entirely otherwise. It causes death sooner or later, and habitually in a period of time varying with the development of the tumor and the site it occupies. Generally the more elevated the tumor, the sooner death occurs: sometimes brought about by the rupture of the sac; at others, from spinal meningitis. Occasionally, the infant falls into coma, and death gradually arises from that direction; but without occupying the time of the society, I will simply add, it is almost necessarily a fatal affection. A few cases of cures are on record; one lately by Chassaignac with iodine injections, recorded in Bonchut's *Maladies des nouveaux nes;* one or two by Sir A. Cooper, referred to in Samuel Cooper's surgical dictionary, treated by puncture and compression, and various other means have been occasionally successful, when the tumor was small and pediculated; but in such a case as this, when the spinal column in the whole lumbar region is nothing more than a gutter formed by the bodies of the lumbar vertebræ, all the appliances of our art must necessarily remain impuissant. This child died from the rupture of the sac. The nurse says it did not sleep from the time it was born until about three o'clock on Thursday afternoon. At five o'clock, she went to it and found it was dead. It seemed to suffer from the moment of birth, making constantly a kind of moaning noise, unlike the cry of a healthy child.

---

